# Characterization of the first complete chloroplast genome of *Amaranthus hybridus* (Caryophyllales: Amaranthaceae) with phylogenetic implications

**DOI:** 10.1080/23802359.2021.1994890

**Published:** 2021-10-27

**Authors:** Xue Bai, Xueling Ye, Yiming Luo, Changyin Liu, Qi Wu

**Affiliations:** Key Laboratory of Coarse Cereal Processing, Ministry of Agriculture and Rural Affairs, Sichuan Province Engineering Technology Research Center of Coarse Cereal Industralization, School of Food and Biological Engineering, Chengdu University, Chengdu, Sichuan, China

**Keywords:** Weed, chloroplast genome, phylogenetic analysis, molecular marker

## Abstract

In the present study, the complete chloroplast genome of *Amaranthus hybridus* was sequenced and assembled. The complete chloroplast genome of *Amaranthus hybridus* is 150,709 in size, with the GC content of 36.56%. The chloroplast genome of *Amaranthus hybridus* contained 86 protein-coding genes (PCGs), eight ribosomal RNA (rRNA) genes, and 37 transfer RNA (tRNA) genes. Phylogenetic analysis based on combined chloroplast gene dataset indicated that the *Amaranthus hybridus* exhibited a close relationship with *A. hypochondriacus* and *A. caudatus*.

The genus *Amaranthus* contains some of the most agronomically important weeds (Montgomery et al. 2020), some of them are used as leafy vegetables, and the others are used for sources of grain or ornamental value (Viljoen et al. 2018). *Amaranthus* is a promising nutritious food source concerning by more and more researchers, just like the tartary buckwheat with high nutritional value (Song et al. 2016; Xiang et al. 2016, 2019a, 2019b). The genus *Amaranthus* comprises approximately 60 species, which are distributed throughout the world (Stetter and Schmid 2017). It is difficult to classify *Amaranthus* species accurately only by morphology. Organelle genomes, including mitochondrial genomes and chloroplast genomes, have been widely used for the phylogeny of eukaryotes (Yang et al. 2019; Li et al. 2019a, 2019b, 2020a; Wang et al. 2020b). So far, the chloroplast genome of *Amaranthus hybridus* has not been sequenced. The complete chloroplast genome of *Amaranthus hybridus* sequenced in this study will promote the understanding of phylogeny and evolution of the genus *Amaranthus*.

The specimen (*Amaranthus hybridus*) was collected from Sichuan, China (102.43E; 31.51N), and then we stored the specimen in Collection Center of Chengdu University (no. ZLX_w7). The complete chloroplast genome of *Amaranthus hybridus* was sequenced and *de novo* assembled according to methods previously described (Li et al. 2018a, 2018b). Briefly, we extracted the total genomic DNA of *Amaranthus hybridus* using a Plant DNA Kit (D3485-00, Omega Bio-Tek, Norcross, GA). Then, the genomic DNA was purified using a Gel Extraction Kit (Omega Bio-Tek, Norcross, GA). The purified DNA was stored in Chengdu University (no. DNA_ ZLX_w7). Sequencing libraries of *Amaranthus hybridus* were constructed using a NEBNext^®^ Ultra™ II DNA Library Prep Kit (NEB, Beijing, China). Whole genomic sequencing (WGS) of *Amaranthus hybridus* was then conducted using the Illumina HiSeq 2500 Platform (Illumina, San Diego, CA). The chloroplast genome of *Amaranthus hybridus* was *de novo* assembled using SPAdes 3.9.0 (Bankevich et al. 2012; Li et al. 2020b). The obtained complete chloroplast genome of *Amaranthus hybridus* was annotated using GeSeq (Tillich et al. 2017).

The complete chloroplast genome of *Amaranthus hybridus* is 150,759 bp in length. The base compositions of the *Amaranthus hybridus* chloroplast genome were as follows: A (31.40%), T (32.04%), G (17.97%), and C (18.60%). The complete chloroplast genome of *Amaranthus hybridus* contains 86 protein-coding genes (PCGs), eight ribosomal RNA (rRNA) genes, and 37 transfer RNA (tRNA) genes (Table S1). To investigate the phylogenetic status of the chloroplast genome of *Amaranthus hybridus*, we constructed a phylogenetic tree for 20 species. The protein coding region of 13 genes conserved in the 20 species was used to construct a combined chloroplast gene set (Wang et al. 2020a, 2020c; Wu et al. 2021). The Bayesian inference (BI) method was used to construct the phylogenetic tree based on combined PCGs of chloroplast genome as described by previous methods (Li et al. 2020c, 2021; Cheng et al. 2021). The chloroplast genome of *Oryza sativa* was used as the outgroup (KM103369). The chloroplast genome of *Amaranthus hybridus* exhibited a close relationship with that of *A. hypochondriacus* and *A. caudatus* (Hong et al. 2019) (Figure 1).

**Figure 1.**
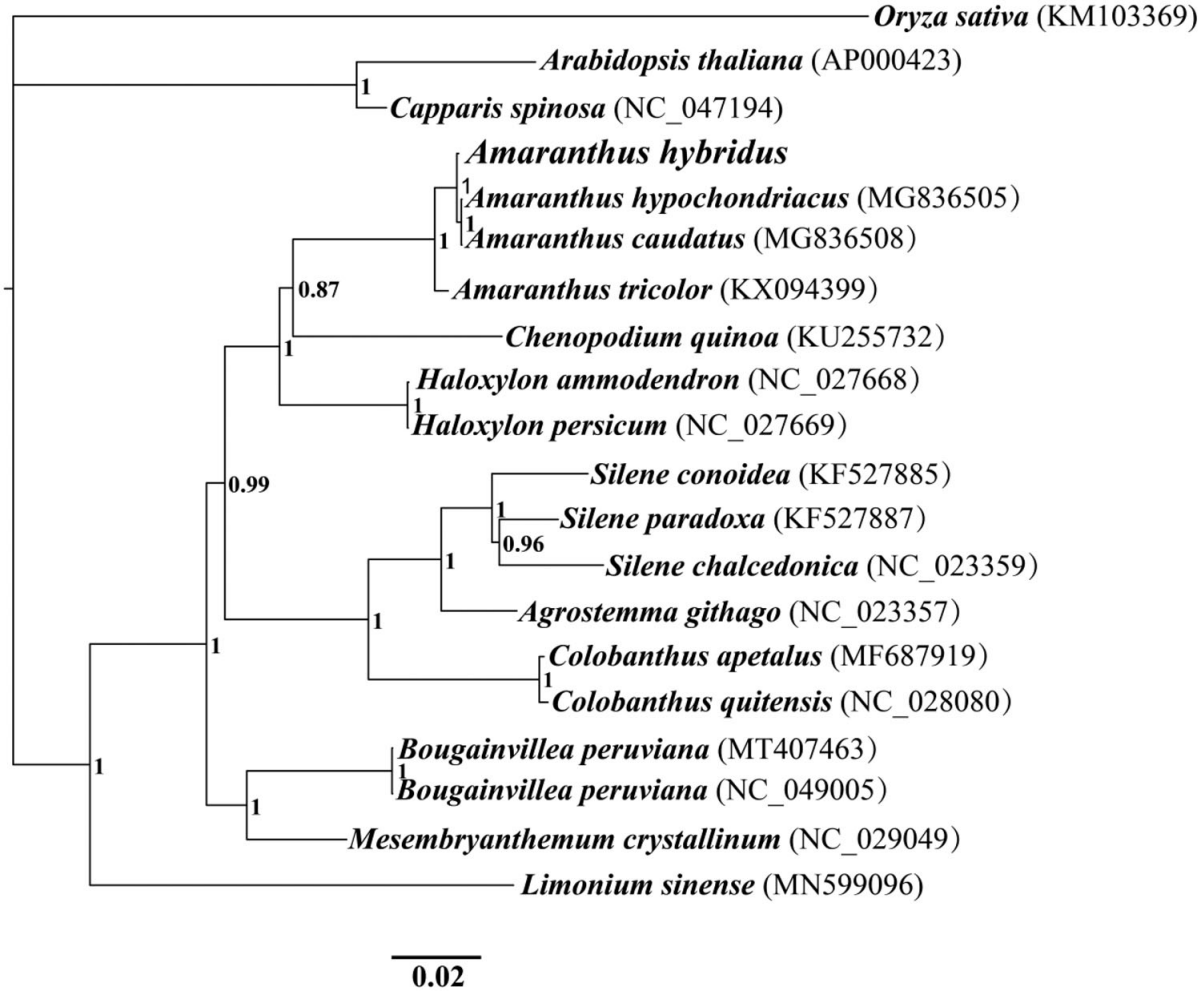
Bayesian phylogenetic analysis of 20 species based on the combined protein-coding genes of chloroplast genome. Accession numbers of chloroplast sequences used in the phylogenetic analysis are listed in brackets after species. Support values are Bayesian posterior probabilities (BPP).

## Supplementary Material

Supplemental Material

## Data Availability

The genome sequence data that support the findings of this study are openly available in GenBank of NCBI at https://www.ncbi.nlm.nih.gov/ under the accession no. MT993471. The associated BioProject, SRA, and Bio-Sample numbers are PRJNA716742, SRR14055740, and SAMN18450400, respectively.
